# Cause-specific mortality at INDEPTH Health and Demographic Surveillance System Sites in Africa and Asia: concluding synthesis

**DOI:** 10.3402/gha.v7.25590

**Published:** 2014-10-29

**Authors:** Osman Sankoh, Peter Byass

**Affiliations:** 1 INDEPTH Network, Accra, Ghana; 2 School of Public Health, Faculty of Health Sciences, University of the Witwatersrand, Johannesburg, South Africa; 3 Faculty of Public Health, Hanoi Medical University, Hanoi, Vietnam; 4 Department of Public Health and Clinical Medicine, WHO Collaborating Centre for Verbal Autopsy, Umeå Centre for Global Health Research, Umeå University, Umeå, Sweden

**Keywords:** mortality, cause of death, Africa, Asia, verbal autopsy, INDEPTH Network

## Abstract

This synthesis brings together findings on cause-specific mortality documented by means of verbal autopsies applied to over 110,000 deaths across Africa and Asia, within INDEPTH Network sites.

**Methods:**

Developments in computerised methods to assign causes of death on the basis of data from verbal autopsy (VA) interviews have made possible these standardised analyses of over 110,000 deaths from 22 African and Asian Health and Demographic Surveillance System sites in the INDEPTH Network. In addition to previous validations of the InterVA-4 probabilistic model, these wide-ranging analyses provide further evidence of the applicability of this approach to assigning the cause of death. Plausible comparisons with existing knowledge of disease patterns, as well as substantial correlations with out-of-model parameters such as time period, country, and other independent data sources were observed.

**Findings:**

Substantial variations in mortality between sites, and in some cases within countries, were observed. A number of the mortality burdens revealed clearly constitute grounds for public health actions. At an overall level, these included high maternal and neonatal mortality rates. More specific examples were childhood drowning in Bangladesh and homicide among adult males in eastern and southern Africa. Mortality from non-communicable diseases, particularly in younger adulthood, is an emerging cause for concern. INDEPTH’s approach of documenting all deaths in particular populations, and successfully assigning causes to the majority, is important for formulating health policies.

**Future directions:**

The pooled dataset underlying these analyses is available at the INDEPTH Data Repository for further analysis. INDEPTH will continue to fill cause-specific mortality knowledge gaps across Africa and Asia, which will also serve as a baseline for post-2015 development goals. The more widespread use of similar VA methods within routine civil registration systems is likely to become an important medium-term strategy in many countries.

This Special Issue on cause-specific mortality has described an unprecedented dataset – more than 110,000 deaths in African and Asian sites, documented over 12 million person-years of population-based surveillance (1). By way of synthesis across the detailed papers, here we reflect on some of the cross-cutting issues in terms of methods, findings, and future directions.

## Methods

In the early years of the INDEPTH Network, following its establishment in 1998, one of the recurring issues reported by sites was the difficulty of getting physicians to assign causes of death to material from verbal autopsy (VA) interviews in a timely, cost-effective, and consistent manner. There is a reason why the vast majority of deaths in African and Asian communities still pass by unregistered and uncertified: usually when someone dies, there is nobody there to certify the death.

There have been over 500 PubMed entries for ‘verbal autopsy’ over the past 25 years, but the past decade has seen rapid developments in methods for handling VA data that do not require a physician being there to give his or her expert assessment of cause of death. Work leading directly to the development of the InterVA-4 model as used here (2) was first published in 2003 (3), from the FilaBavi INDEPTH site in Vietnam.

There have been, and no doubt will continue to be, debates about the advantages and the disadvantages of particular methods and how they relate to what might happen if well-trained and effective physicians were available to assign cause of every death. But at any particular point in time, the relevant question must be ‘what available method can yield adequate information?’ For these analyses, we chose to use the InterVA-4 model (version 4.02) because it was available in the public domain, and complied exactly with the WHO 2012 VA standard (4), and also had an appreciable track record in a number of other studies.

Validation of VA methods is a non-trivial exercise. Physician causes of death assigned to VA material are often considered insufficiently consistent to be regarded as a reference standard for methodological comparisons, so there have been a number of attempts to use harder endpoints such as hospital diagnoses or autopsy findings (5). A Population Health Metrics Research Consortium study of hospital deaths was probably the most extensive of these (6), though it still had methodological problems (7).

Interestingly, though, the application of a particular VA method to a large and wide-ranging dataset also provides further insights into the validity of its outputs. This is particularly the case when patterns that emerge are observed to be linked to parameters that are not part of the VA data processed by the model. Interesting examples here include time period, site, country, altitude, and other independent sources such as the Malaria Atlas Project parasite prevalence map (8). Thus, at the Agincourt, South Africa, site, it was possible to show the complete rise and fall of the overwhelming HIV/AIDS epidemic (9), even though the InterVA-4 model ‘knew’ nothing about the epidemic dynamics. In addition, modelled estimates of cause-specific mortality such as those from international agencies and the Global Burden of Disease (GBD) study (10) can be compared and contrasted, even if the validity of those estimates for countries with sparse data may not be absolute.

InterVA-4 had previously been validated in relation to HIV status in Africa (11) and so it was unsurprising that rates for high-prevalence countries in the HIV/AIDS results from INDEPTH sites here (12) were similar in many cases to UNAIDS and GBD national estimates. Likewise, the dynamics of HIV-related mortality over time – a complete epidemic curve at the Agincourt, South Africa, site and sharp declines from epidemic peaks in other high-prevalence settings – supported the validity with which InterVA-4 was identifying HIV-related deaths. Equally interesting though was the situation in low-prevalence settings. Particularly for the four sites in Bangladesh, very low HIV-related death rates were assigned by InterVA-4 except at the Bandarban site, which covers a militarised high-migration area close to the Myanmar border.

Similarly, analyses in the malaria paper in this Special Issue (13) threw up interesting findings in terms of validity. Using VA to identify possible malaria deaths has long been controversial (14), particularly among adults. Although no biomedical evidence was presented in these analyses, there were significantly higher rates of acute adult febrile deaths (major proportions of which InterVA-4 attributed to malaria) in settings where higher rates of childhood malaria mortality were also attributed, across hundred-fold variations in rates. This is something that can only become clear when VAs are applied systematically and consistently to all deaths in a range of populations, rather than counting local malaria deaths at health facilities or in surveys. The conclusion must either be that there is some proportionality between childhood and adult malaria mortality rates, or, less probably, there is some non-malaria cause of acute adult febrile death that occurs more commonly in high-malaria settings. Equally striking was the significant correlation at the local level between the MAP estimates of parasite prevalence (8) and childhood malaria mortality rates, again across hundred-fold variations. In this case, there was no ambiguity between local findings and national estimates, because the MAP estimates are available in a high-resolution spatial grid from which site areas could be precisely located. These findings, put together, add a considerable sense of validity to InterVA-4’s assignment of malaria as a cause of death.

In totality, this dataset is based on over 20 million individual data items extracted from the original VA interview material, condensed to findings on around 100,000 deaths, and categorised into 60 causes by 22 sites by 14 age–sex groups. Inevitably, there will be both systematic and random errors within this huge amount of material. As with any data processing, the quality of outputs depends on the quality of inputs. Two specific examples of dubious input data led to anomalies: very high rates of digestive neoplasms among adults at the Navrongo, Ghana, site, and almost all deaths from external causes being attributed to transport accidents at the Nouna, Burkina Faso, site. These anomalies may have arisen from issues with historic VA instruments, or with problems in extracting data into the WHO 2012 format. However, in the overall context, these are relatively isolated examples, and certainly do not invalidate the wider findings.

## Findings

Taking all the findings from these large-scale analyses, there were no major surprises, even though some of the burdens of mortality revealed must be regarded as unacceptably high in terms of population health. Some parameters at certain sites varied from national estimates, but there may be good reasons for that. Particularly in the case of Kenya, a highly heterogeneous country in many respects, it was not surprising to find major differences between the coastal site at Kilifi, the site based in the Nairobi slum population, and the inland Kisumu site. One can interpret this either as evidence of the richness of local detail that population surveillance sites can generate or criticise such sites as being individually unrepresentative of any wider situation. This will be an on-going debate for as long as there is no universal civil registration including VA implemented across whole populations. Nevertheless, it is clear, looking across all the sites reported here, that there is considerable diversity in cause-specific mortality, which is being successfully captured.

Across all the sites reported in this supplement, there were examples of mortality burdens that clearly constitute grounds for urgent public health actions – with the advantage that the same tools used in these assessments would be equally applicable for intervention monitoring and evaluation purposes. Some of these mortality burdens were common across all sites – such as high maternal and neonatal mortality rates compared with other parts of the world (15, 16). Other striking findings related to very specific settings and sub-populations, such as childhood drowning in Bangladesh, and homicide among adult males in some eastern and southern African settings (17).

Patterns of non-communicable disease (NCD) mortality are one of the most complex topics explored here (18). NCDs have rapidly acquired an increased importance following the September 2011 UN special high-level meeting and subsequent review meeting in July 2014. Nevertheless, the complex mixture of risk factors, ageing populations, and multiple causes of mortality can make it difficult to separate facts from hyperbole. Consequently, the NCD mortality data presented here distinguish clearly between the premature mortality burden (taken here as under 65 years of age) and the more inevitable occurrence of NCDs as people get older. At this particular point of demographic transition in the low- and middle-income countries represented here, the population proportions of people aged ≥65 are fairly low, but set to increase rapidly. Various risk factors are moving in parallel transitions, with the result that large ‘healthy-exposed’ cohorts are developing, which will influence future NCD mortality. The global concern around NCDs, as being a problem for low-, middle-, and high-income countries alike, is therefore well justified, even if the results presented here suggest that the current premature NCD mortality burden in Africa and Asia is not that high.

An important strength of INDEPTH’s approach to measuring cause-specific mortality is that all deaths in complete populations are included in the surveillance operations, irrespective of health care seeking and other factors. Of course, there will always be a proportion of those deaths which are impossible to follow-up with VA interviews – perhaps because nobody witnessed a death or because nobody was available to respond to the interview. Similarly, there will always be a proportion of VA interviews that contain little or no useful information relating to the cause of death. Nonetheless, including the not-done and indeterminate cases in the analyses is critical to the overall understanding of the burdens related to specific causes of death, as is having all the cause-specific fractions adding up to the total deaths in the population.

## Future directions

Making the pooled dataset (19) publicly available at the INDEPTH Data Repository simultaneously with the publication of this Special Issue is part of the INDEPTH Network’s on-going commitment to open-access data (20). We hope that this will lead to many further analyses based on these important data, beyond the basic descriptions presented in this Special Issue. The INDEPTH Network is also committed to furthering the long-term process of development in VA methods, led by WHO through its Reference Group on Health Statistics and the WHO Collaborating Centre for Verbal Autopsy at Umeå University.

Apart from providing important insights into cause-specific mortality at 22 INDEPTH sites, these analyses also provide clear proof of principle about the viability of large-scale VA operations as the basis for understanding mortality patterns globally. The INDEPTH Network basically exists because of the extremely poor coverage of vital event data across Africa and Asia (21) and would absolutely welcome a scenario in which its operations were no longer relevant because all lives were being systematically documented. Routine application of VA as part of universal civil registration seems the most likely way forward towards that goal, but it is likely to take a long time to achieve anywhere near complete coverage. Although the technical problems are more or less solved, through the WHO 2012 VA standard and models for assigning cause of death like InterVA-4, there remain considerable political and financial challenges. Which ministry or government agency in each country should take the responsibility for implementation? Which budget lines should be used for the necessary resources? How can populations develop confidence that their personal details are being collected for the wider good, rather from any ulterior motive? These are all big questions, which will not find easy answers in many countries.

For the immediate future, therefore, INDEPTH expects to continue its work to fill in some of the information gaps. As the 2015 deadline for the Millennium Development Goals (MDGs) approaches, attention is turning to the post-2015 goals. One of the most problematic areas around evaluating the current MDGs has been the lack of detailed information pertaining to the 1990 starting point. One of the ways in which INDEPTH expects to contribute to the next phase of health-related development goals is by providing a comprehensive set of baseline cause-specific mortality data for the 2012–2015 period. Continued monitoring beyond that baseline, using standardised and comparable methods, will be an important on-going activity for the foreseeable future.

## References

[1] Streatfield PK, Khan WA, Bhuiya A, Alam N, Sie A, Soura AB (2014). Cause-specific mortality in Africa and Asia: evidence from INDEPTH Health and Demographic Surveillance System sites. Glob Health Action.

[2] Byass P, Chandramohan D, Clark SJ, D’Ambruoso L, Fottrell E, Graham WJ (2012). Strengthening standardised interpretation of verbal autopsy data: the new InterVA-4 tool. Glob Health Action.

[3] Byass P, Huong DL, Minh HV (2003). A probabilistic approach to interpreting verbal autopsies: methodology and preliminary validation in Vietnam. Scand J Public Health.

[4] Leitao J, Chandramohan D, Byass P, Jakob R, Bundhamcharoen K, Choprapawon C (2013). Revising the WHO verbal autopsy instrument to facilitate routine cause-of-death monitoring. Glob Health Action.

[5] Leitao J, Desai N, Aleksandrowicz L, Byass P, Miasnikof P, Tollman S (2014). Comparison of physician-certified verbal autopsy with computer-coded verbal autopsy for cause of death assignment in hospitalized patients in low-and middle-income countries: systematic review. BMC Med.

[6] Murray CJ, Lozano R, Flaxman AD, Serina P, Phillips D, Stewart A (2014). Using verbal autopsy to measure causes of death: the comparative performance of existing methods. BMC Med.

[7] Byass P (2014). Usefulness of the Population Health Metrics Research Consortium gold standard verbal autopsy data for general verbal autopsy methods. BMC Med.

[8] Gething PW, Patil AP, Smith DL, Guerra CA, Elyazar IRF, Johnston GL (2011). A new world malaria map: *Plasmodium falciparum* endemicity in 2010. Malar J.

[9] Kabudula CW, Tollman SM, Mee P, Ngobeni S, Silaule B, Gómez-Olivé FX (2014). Two decades of mortality change in rural northeast South Africa. Glob Health Action.

[10] Lozano R, Naghavi M, Foreman K, Lim S, Shibuya K, Aboyans V (2012). Global and regional mortality from 235 causes of death for 20 age groups in 1990 and 2010: a systematic analysis for the Global Burden of Disease Study 2010. Lancet.

[11] Byass P, Calvert C, Miiro-Nakiyingi J, Lutalo T, Michael D, Crampin A (2013). InterVA-4 as a public health tool for measuring HIV/AIDS mortality: a validation study from five African countries. Glob Health Action.

[12] Streatfield PK, Khan WA, Bhuiya A, Hanifi SMA, Alam N, Millogo O (2014). HIV/AIDS-related mortality in Africa and Asia: evidence from INDEPTH Health and Demographic Surveillance System sites. Glob Health Action.

[13] Streatfield PK, Khan WA, Bhuiya A, Hanifi SMA, Alam N, Diboulo E (2014). Malaria mortality in Africa and Asia: evidence from INDEPTH Health and Demographic Surveillance System sites. Glob Health Action.

[14] Butler D (2010). Verbal autopsy methods questioned. Nature.

[15] Streatfield PK, Alam N, Compaore Y, Rossier C, Soura AB, Bonfoh B (2014). Pregnancy-related mortality in Africa and Asia: evidence from INDEPTH Health and Demographic Surveillance System sites. Glob Health Action.

[16] Streatfield PK, Khan WA, Bhuiya A, Hanifi SMA, Alam N, Ouattara M (2014). Cause-specific childhood mortality in Africa and Asia: evidence from INDEPTH Health and Demographic Surveillance System sites. Glob Health Action.

[17] Streatfield PK, Khan WA, Bhuiya A, Hanifi SMA, Alam N, Diboulo E (2014). Mortality from external causes in Africa and Asia: evidence from INDEPTH Health and Demographic Surveillance System sites. Glob Health Action.

[18] Streatfield PK, Khan WA, Bhuiya A, Hanifi SMA, Alam N, Bagagnan C (2014). Adult non-communicable disease mortality in Africa and Asia: evidence from INDEPTH Health and Demographic Surveillance System sites. Glob Health Action.

[19] INDEPTH Network (2014). INDEPTH Network Cause-Specific Mortality – Release 2014. Provided by the INDEPTH Network Data Repository.

[20] Sankoh O, Herbst AJ, Juvekar S, Tollman S, Byass P, Tanner M (2013). INDEPTH launches a data repository and INDEPTHStats. Lancet Glob Health.

[21] Sankoh O, Byass P (2012). The INDEPTH Network: filling vital gaps in global epidemiology. Int J Epidemiol.

